# A minimally-edited mouse model for infection with multiple SARS-CoV-2 strains

**DOI:** 10.3389/fimmu.2022.1007080

**Published:** 2022-11-14

**Authors:** Sandra Nakandakari-Higa, Roham Parsa, Bernardo S. Reis, Renan V. H. de Carvalho, Luka Mesin, Hans-Heinrich Hoffmann, Juliana Bortolatto, Hiromi Muramatsu, Paulo. J. C. Lin, Angelina M. Bilate, Charles M. Rice, Norbert Pardi, Daniel Mucida, Gabriel D. Victora, Maria Cecilia C. Canesso

**Affiliations:** ^1^ Laboratory of Lymphocyte Dynamics, The Rockefeller University, New York, NY, United States; ^2^ Laboratory of Mucosal Immunology, The Rockefeller University, New York, NY, United States; ^3^ Laboratory of Virology and Infectious Disease, The Rockefeller University, New York, NY, United States; ^4^ Department of Microbiology, Perelman School of Medicine, University of Pennsylvania, Philadelphia, PA, United States; ^5^ Acuitas Therapeutics, Vancouver, BC, Canada; ^6^ Howard Hughes Medical Institute, The Rockefeller University, New York, NY, United States

**Keywords:** SARS-CoV-2, mouse ACE2, lung inflammation, mRNA vaccine, memory B cells

## Abstract

Efficient mouse models to study SARS-CoV-2 infection are critical for the development and assessment of vaccines and therapeutic approaches to mitigate the current pandemic and prevent reemergence of COVID-19. While the first generation of mouse models allowed SARS-CoV-2 infection and pathogenesis, they relied on ectopic expression and non-physiological levels of human angiotensin-converting enzyme 2 (hACE2). Here we generated a mouse model carrying the minimal set of modifications necessary for productive infection with multiple strains of SARS-CoV-2. Substitution of only three amino acids in the otherwise native mouse *Ace2* locus (*Ace2* ^TripleMutant^ or *Ace2*™), was sufficient to render mice susceptible to both SARS-CoV-2 strains USA-WA1/2020 and B.1.1.529 (Omicron). Infected *Ace2*™ mice exhibited weight loss and lung damage and inflammation, similar to COVID-19 patients. Previous exposure to USA-WA1/2020 or mRNA vaccination generated memory B cells that participated in plasmablast responses during breakthrough B.1.1.529 infection. Thus, the *Ace2*™ mouse replicates human disease after SARS-CoV-2 infection and provides a tool to study immune responses to sequential infections in mice.

## Introduction

The coronavirus disease 2019 (COVID-19) pandemic, caused by severe acute respiratory syndrome coronavirus 2 (SARS-CoV-2), is an ongoing global health crisis. Rapid development of vaccines against SARS-CoV-2 has been critical to minimizing morbidity and mortality. However, SARS-CoV-2 has shown rapid antigenic drift, which gradually erodes the ability of established antibody responses to control infection. Individuals previously vaccinated or infected with SARS-CoV-2 therefore remain susceptible to emerging viral variants (1–4).

Small animal model systems are key tools in the effort to understand the mechanisms of COVID-19 disease and evaluate therapies and vaccines targeting SARS-CoV-2. However, standard laboratory mice do not support infection with the original USA-WA1/2020 SARS-CoV-2 strain—used in currently available vaccines—due to inefficient interactions between the receptor-binding domain (RBD) of Spike (S) protein and mouse angiotensin-converting enzyme 2 (mACE2) (5, 6). One way to circumvent this limitation has been to develop mouse-adapted strains of SARS-CoV-2 by *in vivo* passaging or reverse genetics (7–10). Conversely, several mouse models that express the full-length human ACE2 (hACE2) protein have been developed, including Ad5-hACE2 (11) or AAV-hACE2 transduced models (12), hACE2 transgenic mice and hACE2 knock-in mice (13–18). A caveat of these mouse models is that the full-length human ACE2 is expressed nonphysiologically and ectopically, altering the tissue or cellular tropism of the virus.

In this study, we sought to establish a mouse model carrying the minimal set of modifications necessary to support infection with multiple strains of SARS-CoV-2. We show that only three amino acid substitutions in the otherwise native mouse *Ace2* locus (*Ace2* ^TripleMutant^ or *Ace2*™) are sufficient to allow infection of mice with both SARS-CoV-2 USA-WA1/2020 (WA-1) strain and the variant B.1.1.529 (Omicron). *Ace2*™ mice can support SARS-CoV-2 WA and Omicron replication in the lungs after intranasal infection, which is accompanied by weight loss and lung damage. Prior exposure to WA-1 infection or vaccination with nucleoside-modified mRNA-lipid nanoparticles (mRNA-LNPs) encoding SARS-CoV-2 spike protein generated memory B cells that participated in plasmablast (PB) responses following Omicron challenge. Our study shows that *Ace2*™ mice are susceptible to all tested variants of SARS-CoV-2, reproducing human pathology after infection and providing a tool to study immune responses to sequential infections in mice.

## Materials and methods

### *In vitro* experiments

Human and mouse ACE2 proteins were expressed in HEK293T cells (purchased from ATCC) after transfection with calcium phosphate. Forty hours after transfection, cells were detached using a non-enzymatic cell dissociation reagent (Thermo Fisher Scientific), washed, and resuspended at 5 x 10^6^ cells per ml in PBE 1x (PBS supplemented with 0.5% BSA + 1 mM EDTA). For each binding reaction, 100 µl of cells were incubated with either recombinant his-tag SARS-COV2 Spike RBD_WA_ or RBD_Omicron_ (Sino Biological) at 3µg/ml, 1µg/ml, 0.3 µg/ml or no protein at 4°C for 30 minutes. Cells were washed two times with PBE 1x and the bound protein was detected with a His-tag mAb APC-conjugated (Cell Signaling Cat. No. 14931S) by flow cytometry. Experiments were performed twice with three wells per condition each time.

### Cell lines

VeroE6 cells (*Chlorocebus sabaeus*; sex: female, kidney epithelial) obtained from ATCC (CRL-1586™) and from Ralph Baric (University of North Carolina at Chapel Hill), and Caco-2 cells (*Homo sapiens*; sex: male, colon epithelial) obtained from the ATCC (HTB-37™) were cultured in Dulbecco’s Modified Eagle Medium (DMEM) supplemented with 1% nonessential amino acids (NEAA) and 10% fetal bovine serum (FBS) at 37°C and 5% CO_2_. All cell lines tested negative for contamination with mycoplasma.

### Virus and virus titration

SARS-CoV-2 strains USA-WA1/2020 (WA-1) and the variant B.1.1.529 sublineage BA.1 (Omicron) were obtained from BEI Resources (catalog no. NR-52281 and NR-56461, respectively). WA virus was amplified in Caco-2 cells, which were infected at a multiplicity of infection (MOI) of 0.05 plaque forming units (PFU)/cell and incubated for 5 days at 37°C. The Omicron variant was amplified in VeroE6 cells obtained from ATCC that were engineered to stably express TMPRSS2 (VeroE6_TMPRSS2_). VeroE6_TMPRSS2_ cells were infected at a MOI = 0.05 PFU/cell and incubated for 4 days at 33°C. Virus-containing supernatants were subsequently harvested, clarified by centrifugation (3,000 g × 10 min), filtered using a disposable vacuum filter system with a 0.22 μm membrane, and stored at -80°C. Virus stock titers were measured by standard plaque assay on VeroE6 cells obtained from Ralph Baric (referred to as VeroE6_UNC_). Briefly, 500 µL of serial 10-fold virus dilutions in Opti-MEM were used to infect 4x10^5^ cells seeded the day prior into wells of a 6-well plate. After 1.5 h adsorption, the virus inoculum was removed, and cells were overlayed with DMEM containing 10% FBS with 1.2% microcrystalline cellulose (Avicel). Cells were incubated for 4 days at 33°C, followed by fixation with 7% formaldehyde and crystal violet staining for plaque enumeration. All SARS-CoV-2 experiments were performed in a biosafety level 3 laboratory (BSL3).

### Experimental mice

All mice used in this study were maintained under specific pathogen-free conditions at the Rockefeller University Comparative for Biosciences Center. All experimental procedures involving SARS-CoV-2 infection were conducted BSL3 and approved by the Rockefeller University’s Institutional Animal Care and Use Committee (IACUC protocol number 19033H). Wild-type C57BL/6J and Ai14 (*Rosa26* ^Lox-Stop-Lox-tdTomato^) mice were obtained from the Jackson Laboratory. *S1pr2^CreERT2^* BAC-transgenic mice (19) were generated and kindly provided by T. Kurosaki and T. Okada (Osaka University and RIKEN-Yokohama). *Ace2*™ mice were generated following two rounds of the EASI-CRISPR protocol to first insert mutation H353K and later S82M/F83Y (20). Briefly, fertilized C57BL/6J zygotes at the one-cell stage were cytoplasmically injected with Cas9 protein, sgRNA targeting *Ace2* exon 9 or exon 3 and the corresponding repair ssDNA template for mutation H353K and S82M/F83Y, respectively. The resulting mice were genotyped using primers Exon9_F (tgcagaaaggatatttcaagaggcag) and Exon9_R (atgttctcccttggacttccagtc) followed by AvaI (NEB) digestion; and primers Exon3_F (aggactaagccatgcaggaagtag), Exon3_R (tcagtgctgaccatggtgtagcag) followed by digestion with TatI (Thermo Fisher Scientific). Single-targeted *Ace2* ^H353K^ and *Ace2* ^S82M/F83Y^ mice were generated as byproducts of the double-targeting process. For fate-mapping experiments, *Ace2* ^TM/TM^ females were crossed with *R26* ^Tom/Tom^ homozygous *S1pr2* ^CreERT2^ transgenic males. Male and female littermates 8-14 weeks old were used for all experiments and mice were randomly distributed to cages. Because of the high chance of contamination, it was not possible to house mice infected with different variants or non-infected mice together. A minimum of biological triplicates was used throughout the study. For weight loss measurements a total of 5 to 16 mice per group were analyzed; for qPCR and histological score measurements a total of 3 to 6 mice per group were analyzed; for longitudinal analysis of lung immune response to B.1.1.529 sublineage BA.1, a total of 3 mice per group were analyzed; for fate-mapping experiments a total of 4 to 5 mice per group were analyzed. Blinding was not possible in this study as the experimenters treating the mice were the same as those that analyzed the data. The treatment groups had to be clearly identified throughout the study to prevent cross contamination.

### Infection models, vaccination and fate-mapping

8–14-week-old mice were anesthetized with ketamine/xylazine diluted in sterile PBS 1x (Gibco, Inc.) and infected intranasally with 2.34x10^5^ PFU in 30 μl of SARS-CoV-2 strain USA-WA1/2020 (WA-1) (P3) or 3.3x10^5^ PFU in 30 ul of variant B.1.1.529 sublineage BA.1 (P2). Membrane-bound full-length Spike (S) protein mRNA-LNPs with a proline substitution in the S2 subunit (K986P and V987P) to stabilize the prefusion conformation were designed using the full S protein sequence of SARS-CoV-2 Wuhan-Hu-1 strain (GenBank MN908947.3) as previously described (21). mRNA was produced and encapsulated in LNPs as previously reported (22). Mice were immunized in the gastrocnemius muscle with 30 μg mRNA-LNP. Fate-mapping of germinal centers (GCs) in S1pr2-Tomato mice was carried out by intragastric administration of two doses of 2.5 mg of tamoxifen citrate (Sigma Aldrich) diluted in corn oil at day 7 and day 14 post-infection or vaccination (10mg/ml). Weight loss was assessed until day 6 post infection and results show a pool of three independent experiments (n = 4, 3, 8 per experiment for *Ace2*™+WA-1 group; n = 3, 3, 5 per experiment for *Ace2*™+Omicron group; n = 3, 3, 3 per experiment for WT+Omicron group, n = 3, 2 per experiment for WT+WA-1 group). For lungs of WT and *Ace2*™ mice were dissected 3 days post infection and equally distributed for qPCR and histology analysis. Results show a representative of two independent experiments (n = 3, 3 per experiment for *Ace2*™+WA-1 group; n = 3, 3 per experiment for *Ace2*™+Omicron group; n = 3, 3 per experiment for WT+Omicron group, n = 3, 3 for WT+WA-1 group. Lungs and mediastinal lymph nodes of *Ace2*™ mice were dissected at different time points post Omicron infection, as indicated in the figure, and digested for flow cytometry analysis (n = 3 mice per group for each time point). *Ace2*™ S1pr2-Tomato mice were infected with WA-1 or vaccinated with S-encoding mRNA-LNPs, followed by tamoxifen treatment 7 and 14 days later. Infected and vaccinated animals were then subjected to breakthrough infection with Omicron virus 4 weeks after the primary exposure. Serum was collected before infection or vaccination (week 0) and at weeks 1, 2 and 4 post primary infection or vaccination and 10 days after Omicron infection. Mediastinal lymph nodes were collected for flow cytometry analysis 10 days post Omicron infection. Results show a pool of two independent experiments (n = 3, 1 per experiment for WA-1 group; n = 3, 1 per experiment for WA-1+Omicron group; and n = 3, 2 per experiment for mRNA+Omicron).

### Tissue preparations

Disposable micropestles (Axygen) were used to mechanically disassociate LNs into cell suspensions. For lung preparation, to identify blood contamination during flow cytometer analysis mice were first intravenously injected with 3 µg of anti-mouse CD45-FITC (Fisher Scientific Cat. No. 553080) 3 minutes before euthanasia by exposure to high dose isoflurane. All lung lobes were dissected and distributed for qPCR, flow cytometer and histology analysis, roughly 1/3 of the lobes for each assay. For qPCR, the lung lobes were homogenized in 3 ml of Trizol (Invitrogen) using a gentleMACS Dissociator (Miltenyi Biotec). Total RNA was extracted with phenol-chloroform followed by RNeasy Mini kit (Qiagen). Viral RNA titers were calculated by RT-qPCR targeting the S gene of SARS-CoV-2 with primers SARS-CoV-F (tcctggtgattcttcttcaggt) and SARS-CoV-R (tctgagagagggtcaagtgc) (15) using SYBR Green Real-Time PCR master mix (Thermo Fisher Scientific). For histology, the lung lobes were fixed in 4% paraformaldehyde for further paraffin tissue preparation. For flow cytometry, the lung lobes were first cut into smaller pieces and then digested in RPMI solution containing 1.5 mg/ml Collagenase A (Sigma-Aldrich) and 1 mg/ml Dnase I (Roche) for 45 min at 37°C and 80 rpm shaking. Next, the digested tissue was transferred through a 70 µm nylon mesh and immune cells were isolated by gradient centrifugation (underlayer 40% and top layer 80% containing cells) using Percoll. Mediastinal LNs (mLNs) were harvested and mechanically dissociated into cell suspensions using disposable micropestles (Axigen).

### Flow cytometry

Cells from each tissue were resuspended in PBE 1x (PBS supplemented with 0.5% BSA + 1 mM EDTA) and incubated for 30 min on ice with fluorescently labeled antibodies: CD45-AF700 (BioLegend Cat. No. 103127), CD4-BUV495 (BD Biosciences Cat. No. 565974), CD8α-BUV805 (BD Biosciences Cat. No. 612898), TCRβ-BUV395 (BD Biosciences Cat. No. 742485), NK1.1-BV785 (BioLegend Cat. No. 108749), CD11b-BV711 (BioLegend Cat. No. 101241), CD11c-BUV496 (BD Biosciences Cat. No. 750483), I-A/I-E-BUV395 (BD Biosciences Cat. No. 743876), Ly6G-BV605 (BioLegend Cat. No. 127639), F4/80-BV785 (BioLegend Cat. No. 123141), IFN-δ-PerCP-Cy5.5 (BD Biosciences Cat. No. 560660), TNF-α-PE-Cy7 (BioLegend Cat. No. 506323), IL-17α-BV421 (BioLegend Cat. No. 506925), B220-BV785 (BioLegend Cat. No. 103245), FAS-PE-Cy7 (BioLegend Cat. No. 152617), CD38- PerCP-Cy5.5 (BioLegend Cat. No. 102721) and CD138-BV650 (BioLegend Cat. No. 142517). WA-1 RBD and Omicron RBD biotinylated were purchased from Sino Biological and conjugated with streptavidin. LIVE/DEAD Fixable Aqua Dead Cell Stain Kit, L-34965, was purchased from Life Technologies. Cells were filtered and washed with PBE 1x again before analysis on BD FACS Symphony cytometer. Analyses were performed using FlowJo v. 10 software.

### ELISA

RBD_WA-1_ and RBD_Omicron_-specific antibodies were measured by ELISA using 1µg/ml of recombinant his-tag SARS-COV2 Spike RBD_WA-1_ or RBD_Omicron_ (Sino Biological), respectively. Serum was assayed in 3-fold dilutions starting at 1/100 and total IgG antibodies were detected using goat anti-mouse IgG conjugated to horseradish peroxidase and developed with tetrametghylbenzidine (Sigma). OD450 was measured with an accuSkan FC microplate photometer (Fisher Scientific) and antibody titers were calculated by logarithmic interpolation of the dilutions with readings immediately above and immediately below an OD450 of 0.1.

### Histological score

15 µm-thick sections were stained with hematoxylin and eosin (H&E) and imaged using a brightfield microscope (Keyence, BZ X-810). Histological scoring was done according to a previous publication (7). Briefly, images were scored from 0-12 based on the presence of interstitial congestion, epithelial damage, inflammatory infiltrate, peribronchiolar lymphatic inflammation, hemorrhage, and intrabronchial macrophages. Tissue pathology scores were performed blinded and samples were scored for each feature as 0 (absence), 1 (low prevalence) or 2 (high prevalence).

### Statistical analyses

Statistical tests used to compare conditions are indicated in the figure legends. Statistical analysis was carried out using GrahPad Prism v.9. Flow cytometry analysis was carried out using FlowJo v.10 software. Graphs were plotted using Prism v.9 and edited for appearance using Adobe Illustrator CS. Comparisons between two treatment conditions were analyzed using two-tailed Student’s t-test. Multivariate data were analyzed by applying one-way ANOVA and Tukey’s multiple comparison *post hoc* test. Mixed-effects analysis followed by Tukey’s multiple comparison test was used for weight loss experiments. Statistical details of experiments are provided in the results, figures, and corresponding figure legends. A *P*-value of less than 0.05 was considered significant.

## Results

### Development of a mouse model that supports infection with different variants of SARS-CoV-2

**Figure 1:**
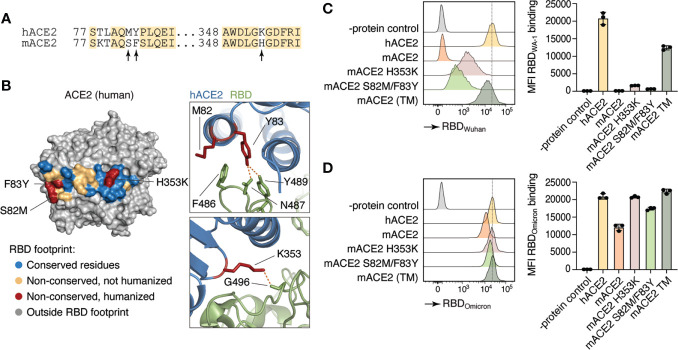
Differences in human and mouse ACE2 protein structure and SARS-CoV-2 Spike binding capacity. **(A)** Amino acid sequence of human ACE2 (hACE2) and mouse ACE2 (mACE2). Arrows indicate amino acid residues involved in SARS-CoV-2 Spike binding site. **(B)** Crystal structure of human ACE2 (23). Colored residues denote the SARS-CoV-2 S binding interface (inter-chain distance <5.0 Å). Residues conserved between human and mice are shown in blue. Non-conserved residues are shown in yellow and red, the latter indicating the three positions humanized in *Ace2*™. **(C, D)** Human (hACE2), mouse (mACE2) or mutant mouse ACE2 (H353K, S82M/F83Y, or TM) proteins were expressed in HEK293T cells and incubated with APC-labeled recombinant WA-1 **(C)** or Omicron **(D)** RBDs. Binding capacity was measured by flow cytometry. Histogram and MFI quantification are depicted. Dotted line shows hACE2-RDB binding MFI as a reference. HEK293T cells expressing an irrelevant protein were used as controls.

Small animal models that recapitulate the progression of SARS-CoV-2 pathogenicity in humans are critical for advancing COVID-19 knowledge. However, SARS-CoV-2 does not use mACE2 as its receptor for cellular entry and infection (5). To first test whether we could increase mACE2 binding to SARS-CoV-2 by substituting a limited number of amino acids with their human counterparts, we introduced amino acid substitutions previously reported to be important for binding to SARS-CoV-2 spike protein in two sites of the murine *Ace2* cDNA (**Figure 1A**). The first was a His to Lys substitution at position 353 (H353K), a residue at the core of the ACE2–RBD interaction which has been shown to allow replication of the original SARS coronavirus in mouse cells (24), and the second was a Phe to Tyr substitution at position 83 (F83Y), which makes multiple direct contacts with the SARS-CoV-2 RBD (25) (**Figure 1B**). Because of its proximity to F83 and of the nonconservative nature of the replacement, we also humanized neighboring position 82 by introducing a Ser to Met substitution (S82M). We transfected HEK293T cells with versions of mACE2 carrying S82M/F83Y, H353K, or all three mutations ™ and tested their ability to bind to the WA-1 and Omicron RBDs. mACE2 binding to RBD_WA-1_ was partially rescued to hACE2 levels by either the H353K or the S82M/F83Y substitutions, whereas mACE2 TM restored binding to WA-1 RBD to comparable levels to that of hACE2 (**Figure 1C**). In line with other groups showing mouse susceptibility to Omicron infection (26), we observed substantial mACE2 binding to RBD_Omicron_. Nevertheless, introduction of all three mutations doubled the binding of mACE2 to hACE2 levels (**Figure 1D**).

**Figure 2:**
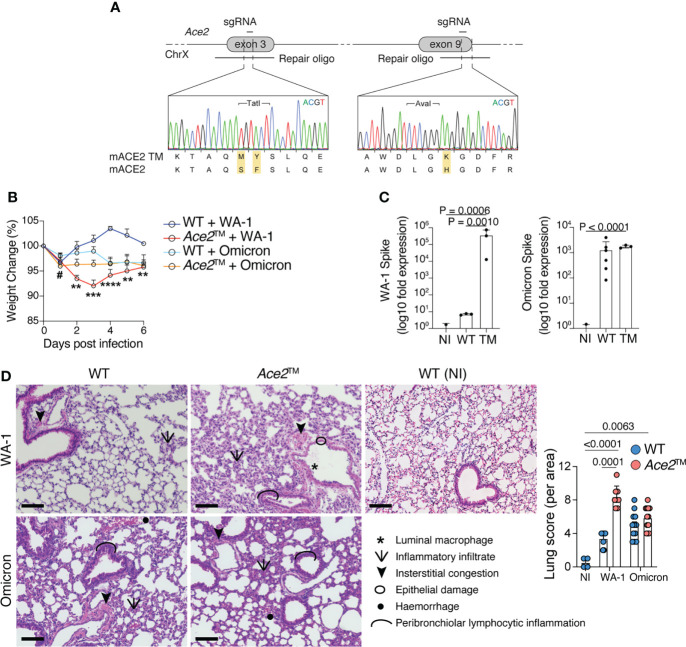
*Ace2*™ mice are susceptible to different variants of SARS-CoV-2. **(A)** Design of the *Ace2*™ allele, depicting the double S82M/F83Y replacement in exon 3 and the H353K replacement in exon 9 of mouse *Ace2*, with representative chromatograms obtained by Sanger sequencing of targeted mice. **(B–D)** 8-14-week-old wild-type (WT) and *Ace2*™ mice were infected with WA-1 (2.34x10^5^ PFU) or Omicron (3.3x10^5^ PFU) strains. **(B)** Percent change in body weight in different experimental groups (n = 5 to 16 mice per group, pool of three independent experiments). **(C)** qRT-PCR of WA-1 *Spike* (left) and Omicron *Spike* (right) RNA loads in the lungs of mice at 3 dpi, shown as fold-change over background levels in non-infected (NI) control mice. Total RNA input was normalized prior to qRT-PCR analysis (n = 3 to 6 mice per group, representative of two independent experiments). **(D)** Pathological scores of lung specimens from NI control mice or WA-1 and Omicron infected *Ace2*™ and WT mice. Tissue pathology was assessed based on the presence of the indicated features in distinct areas from each lung specimen (n = 3 mice per group). Data were analyzed by one-way ANOVA. Scale Bar = 100 μm. ^#^p ≤ 0,05 comparison between *Ace2*™ and WT animals infected with B.1.1.529 1 dpi; **p ≤ 0.01, ***p ≤ 0.001, ****p ≤ 0.0001 comparison between *Ace2*™ and WT animals infected with USA-WA1/2020. Data were analyzed by mixed-effects analysis followed by Tukey’s multiple comparison test.

We next developed a genetically engineered mouse line by inserting these three mutations into the endogenous mouse *Ace2* locus. Using two rounds of CRISPR/Cas9 targeting, we edited *Ace2* exons 3 and 9 with 2 different sgRNAs and their corresponding repair templates (**Figure 2A**). To evaluate whether the *Ace2*™ allele was sufficient to allow infection with SARS-CoV-2, wild-type C57BL/6 (WT) mice and *Ace2*™ mice were infected intranasally with WA-1 or Omicron strains of SARS-CoV-2. *Ace2*™ mice infected with WA-1 exhibited significant weight loss starting at 2 days post infection (dpi), whereas no weight loss was observed for WT mice infected with this strain (**Figure 2B**). In agreement with the *in vitro* data, *Ace2* ^H353K^ single mutant mice and *Ace2* ^S82M/F83Y^ double mutant mice infected with WA-1 did not exhibit significant changes in body weight (**Supplementary Figure 1**). When infected with Omicron, *Ace2*™ mice exhibited a transient decrease in body weight at 1 dpi that was only slightly more severe than that experienced by similarly infected WT mice (**Figure 2B**). To confirm that *Ace2*™ mice were indeed infected with each strain, we harvested the lungs of WT and *Ace2*™ mice at 3 dpi and performed PCR for viral RNA. *Ace2*™ mice showed a >100,000-fold increase in WA-1 and a >1,000-fold increase in Omicron viral RNA signal over background levels seen in non-infected mice (**Figure 2C**). Of note, WT mice showed similar lung viral loads to *Ace2*™ mice when infected with Omicron, but much lower viral RNA levels when infected with WA-1.

Histopathologic examination of lung sections harvested at 3 dpi showed that *Ace2*™ mice infected with WA-1 displayed increased peribronchiolar lymphocytic inflammation, luminal macrophages, hemorrhage, and epithelial damage in the lung when compared to WT mice (**Figure 2D** and **Supplementary Figure 2**). Both WT and *Ace2*™ mice infected with Omicron displayed mild diffuse peribronchial infiltrates and hemorrhage (**Figure 2D**), consistent with the lower titers of viral RNA and weight loss caused by Omicron infection. Together, these data show that *Ace2*™ mice support infection and develop disease with both WA-1 and Omicron strains of SARS-CoV-2.

### The immune response to Omicron B.1.1.529 infection

**Figure 3:**
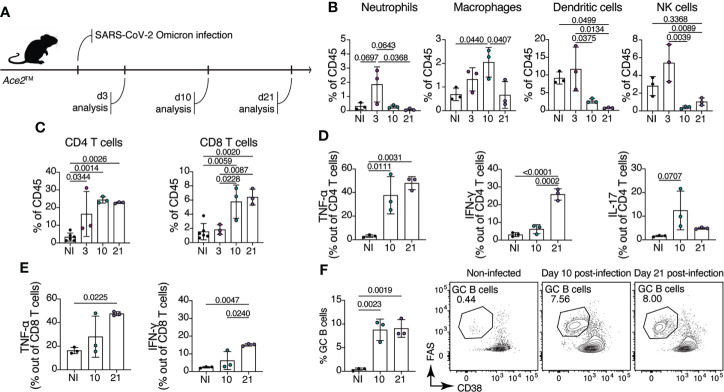
Immune response of *Ace2*™ mice to B.1.1.529 infection. **(A-F)** 8-14-week-old *Ace2*™ mice were infected with Omicron and lung cells were harvested for flow cytometry at 3, 10, and 21 dpi. **(A)** Schematic of the infection and analysis protocol. **(B)** Frequency of neutrophils (CD11b^+^Ly6G^+^), macrophages (CD11b^+^F4/80^+^), dendritic cells (MHCII^+^CD11c^+^) and NK cells (NK1.1^+^) among total lung CD45^+^ cells from infected animals. **(C)** Frequency of CD4^+^ (TCRβ^+^CD4^+^) and CD8^+^ (TCRβ^+^CD8a^+^) T cells among total lung CD45^+^ cells. **(D, E)** Lung cells isolated at 10 and 21 dpi were stimulated with PMA and ionomycin for 4 hours prior cytokine staining. **(D)** Frequency of TNF-α^+^ (left), IFN-γ^+^ (center) and IL-17^+^ (right) CD4^+^ T cells. **(E)** Frequency of TNF-α^+^ (left), IFN-γ^+^ (right) CD8^+^ T cells. **(F)** Frequency of germinal center B cells (CD38^low/–^Fas^hi^) in the mediastinal lymph node (mLN) of infected animals at 10 and 21 dpi (n = 3 mice per group). Data were analyzed by one-way ANOVA.

To evaluate the composition of the immune cell response in *Ace2*™ mice infected with Omicron, we performed flow cytometric analysis on lung homogenates at days 3, 10 and 21 after intranasal virus inoculation (**Figure 3A**). We observed an infiltration of innate immune cells in the lung of *Ace2*™ mice, characterized mainly by Ly6G^+^ neutrophils, F4/80^+^ macrophages, MHCII^+^CD11c^+^ dendritic cells and NK1.1^+^ natural killer cells at 3 dpi (**Figure 3B**). The frequencies of these cells decreased by 10 dpi, with the exception of macrophages, which were still present in the lungs at this timepoint. By 10 dpi, we also observed an accumulation of CD4^+^ and CD8^+^ T cells in the lungs, which was maintained until 21 dpi (**Figure 3C**). Progression and severity of COVID-19 disease is associated with changes in cytokine profile (27, 28). Accordingly, we observed an increase in TNF-α and IL-17 production by CD4^+^ T cells 10 dpi, whereas IFN-γ production by these populations began later, at around 21 dpi (**Figure 3D**). Production of TNF-α and IFN-γ by CD8^+^ T cells followed a similar temporal trend (**Figure 3E**).

Antibodies specific for the SARS-CoV-2 spike (S) protein, and especially those binding the RBD, are important for controlling and preventing reinfection. Antibody producing cells are formed in germinal centers (GCs), specialized microstructures in secondary lymphoid tissues that harbor B cell proliferation and antibody affinity maturation (29). To assess the formation of GCs upon Omicron infection in *Ace2*™ mice, we isolated mediastinal lymph nodes (mLN) from infected animals at day 10 and 21 post-infection. *Ace2*™ mice infected with Omicron displayed large GCs (CD38^low/-^ Fas^hi^ B cells) at 10 dpi, which accounted for an average of 8.7% of all B cells in the mLN. GC size was maintained at least through 21 dpi (**Figure 3F**). These data show that, despite mild histopathological damage, robust innate and adaptive immune responses are elicited in the lungs of *Ace2*™ mice infected with Omicron.

### Memory B cell dynamics in breakthrough Omicron infection

**Figure 4:**
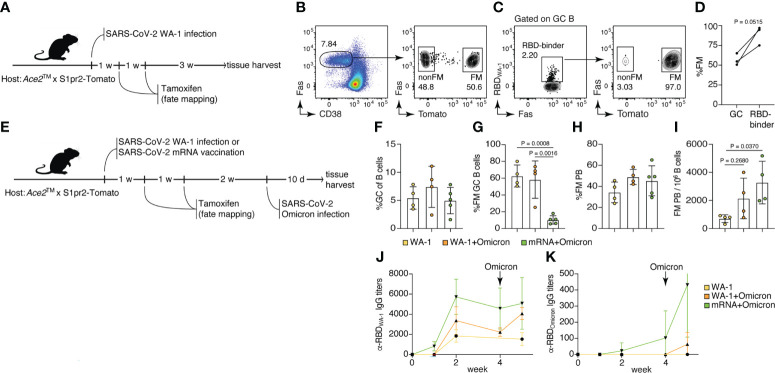
Memory B cells generated by USA-WA1/2020 infection or mRNA vaccination are preferentially recruited to the plasmablast compartment upon B.1.1.529 breakthrough infection. **(A–D)** 8-14-week-old *Ace2*™ mice were infected with WA-1 SARS-Cov-2 strain, and GC B cells were fate-mapped (FM) by tamoxifen administration at 7 and 14 dpi. **(B)** Frequency of FM cells among total GC B cells. **(C, D)** Frequency of FM cells among WA-1 Spike RBD binders. **(E–K)** *Ace2*™ mice were either infected with WA-1 or vaccinated with SARS-CoV-2 S protein-encoding mRNA-LNP, fate-mapped, then and challenged with Omicron variant 4 weeks later. **(F)** Frequency of GC B cells among total B cells; **(G)** frequency of FM cells among GC B cells; **(H)** frequency of FM cells among PB; and **(I)** frequency of FM PB per million B cells in the WA-only infected group (yellow), WA+Omicron reinfection group (orange), and mRNA+Omicron challenge group (green). **(J)** WA-1 RBD-specific and **(K)** Omicron RBD-specific serum IgG antibody titers among groups (n = 2, 3 mice per group, pool of two independent experiments). Data were analyzed by t-test or one-way ANOVA.

Rapid mutations in SARS-CoV-2 have resulted in increased susceptibility to new variants of the virus, especially of the Omicron lineage, in individuals previously vaccinated or infected with other variants of SARS-CoV-2. Using the *Ace2*™ model, we sought to determine how memory B cells elicited by WA-1 infection or mRNA vaccination respond to breakthrough infection with Omicron. To label memory cells generated by primary exposure, we crossed *Ace2*™ mice with mice carrying the S1pr2-CreERT2 BAC-transgenic driver and the *Rosa26* ^Lox-Stop-Lox-tdTomato^ reporter (S1pr2-Tomato), a combination that allows highly efficient fate-mapping of GC B cells and their progeny by tamoxifen administration (19). To test our ability to fate-map GC B cells responding to WA-1 infection, we infected *Ace2*™ S1pr2-Tomato mice with WA-1 and administered tamoxifen at 7 and 14 dpi (**Figure 4A**). Five weeks post-infection, ~56.7% of all GC B cells (and 88.9% of RBD-binding cells) in mLNs expressed tdTomato, indicating that fate-mapping is able to enrich for GC B cells responding to the primary infection (**Figures 4B–D**).

To investigate the recruitment of memory B cells elicited by primary SARS-CoV-2 infection or vaccination by secondary Omicron challenge, we either infected or vaccinated *Ace2*™ S1pr2-Tomato mice with WA-1 or S-encoding mRNA-LNPs, followed by tamoxifen treatment 7 and 14 dpi. Infected and vaccinated animals were then subjected to breakthrough infection with Omicron virus 4 weeks after the primary exposure (**Figure 4E**). GC size across all three groups (WA-1 only, WA-1+Omicron and mRNA+Omicron) remained similar, indicating that Omicron challenge did not induce a significant expansion of the GC compartment (**Figure 4F**). The WA-1+Omicron reinfection group had similar percentages (~58.3%) of fate-mapped cells to the WA-1-only infected group (~62.6%) in mLN GCs, suggesting efficient maintenance of fate-mapped cells in the GC following challenge and inefficient recruitment of new B cell clones into the ongoing GCs after Omicron breakthrough infection (**Figure 4G**). When breakthrough infection followed mRNA vaccination, recruitment of fate-mapped memory B cells generated in the vaccine-draining lymph node (inguinal LNs) into the mLN GCs was inefficient (~10.3% in the mRNA+Omicron group; **Figure 4G**). Fate-mapped cells were present in the plasmablast (PB) compartment at similar frequencies in all 3 groups (**Figure 4H**); however, the total number of fate-mapped PB cells upon breakthrough Omicron infection was increased in previously vaccinated or WA-1-infected mice compared to unexposed controls (**Figure 4I**). Together, these observations support a model in which, upon challenge, memory B cells are preferentially recruited to the antibody-secreting plasma cell compartment rather than reentering secondary GCs (30). This is further supported by the increase in RBD_WA-1_-specific IgG serum antibody titers after Omicron infection in the vaccination and reinfection groups, compared to the WA-1-only group (**Figure 4J**). Interestingly, RBD_Omicron_-specific serum antibodies were detected in the mRNA+Omicron group even prior to Omicron challenge, but in the WA-1-infected group only after exposure to the Omicron variant, and at lower levels (**Figure 4K**). We conclude that WA-1 infection and S protein mRNA vaccination generate cross-reactive memory B cells that are reengaged by breakthrough Omicron infection, recapitulating findings in the human system (31).

## Discussion

The COVID-19 pandemic has raised an urgent need for novel drugs and therapeutics aimed at preventing infection and severe disease. Achieving this requires animal models that recapitulate human COVID-19 disease progression and innate and adaptive immune responses. In this study, we developed one such model by humanizing three amino acid positions of the mouse *Ace2* gene (*Ace2*™). This model supports infection by at least two isolates of SARS-CoV-2, USA-WA1/2020 (WA-1) and variant B.1.1.529 (Omicron), as well as lung damage and inflammatory infiltrate, reproducing the effects of COVID-19 disease in humans. Higher morbidity and mortality rates have been consistently observed in humans exposed to the original SARS-CoV-2 strain when compared to the those exposed to Omicron variants, which cause milder respiratory infection (32). *Ace2*™ mice recapitulate this feature, as mice infected with WA-1 exhibit greater weight loss and more severe lung inflammation when compared to mice infected with Omicron. Even though the effects of Omicron infection are milder in *Ace2*™ mice, infection still elicits a robust immune response characterized by neutrophil and macrophage infiltration in the lungs in early infection, followed by CD4^+^ and CD8^+^ T cells producing TNF-α and IFN-γ and formation of GCs in the mLN. We did not observe mortality related to either infection, again in line with the typical outcome of SARS-CoV-2 infection in healthy humans.

Although C57BL/6 WT mice cannot be infected by WA-1-type viruses (5, 6), Omicron variants, similarly to other variants of SARS-CoV-2 that emerged throughout the pandemic, acquired a mouse-adapting S substitution (N501Y) that allows viral infection to proceed through mouse ACE2 (26, 33–35). In accordance with other studies (26, 36), our data show that C57BL/6 WT mice are susceptible to Omicron infection at similar levels as the *Ace2*™ mice, although virus titers are slightly higher in the latter. An advantage of the *Ace2*™ model is that these mice are susceptible to both WA-1 and Omicron variant, allowing us to study omicron breakthrough reinfection after WA-1 infection or mRNA-vaccination, a common sequence of events in the human COVID-19 pandemic (37, 38).

As previously reported in other models, memory B cells generated at a distal site (in this study at inguinal LNs after vaccination) made up a small percentage of secondary GCs, which were composed primarily of B cells of naïve origin (30). The increased participation of memory cells in the PB compartment for both the WA-1+Omicron and mRNA+Omicron groups compared to WA-1-only infection indicates a robust antibody response to a new challenge even in the presence of an ongoing response. Taken together, our results support a model in which memory B cells preferentially become antibody-secreting cells after exposure to related antigens. Whether these memory-derived antibodies are protective against a new challenge and the extent of their contribution in the total antigen-specific serum antibody pool still needs to be elucidated.

A variety of mouse models have been previously developed to render mice susceptible to SARS-CoV-2 infection by expression of hACE2 *via* genomic transgenes or knock-in alleles (13–17) or viral vectors (11, 12). Although these studies have made significant contributions to our understanding of SARS-CoV-2 infection, some limitations related to the immunogenicity and expression kinetics of AdV and AAV should be considered, especially when studying the intensity and duration of the immune response and repeated infections over time. Another caveat with some of the transgenic mice expressing hACE2 is the high number of hACE2 transgene insertions as well as ectopic expression of the gene, which can change tissue and cellular tropism of the virus. One example is the SARS-CoV-2 infection in the brain of K18-hACE2 transgenic mouse and the high levels of mortality observed in this mouse model (39, 40). The three mutations we used here to develop the *Ace2*™ mouse model represent the minimum alterations necessary to make mice fully susceptible to all tested variants of SARS-CoV-2 without affecting the organization of the mouse *Ace2* gene.

## Data availability statement

The raw data supporting the conclusions of this article will be made available by the authors, without undue reservation.

## Ethics statement

The animal study was reviewed and approved by Institutional Animal Care and Use Committee (IACUC).

## Author contributions

SN-H and MCCC conducted the majority of the experiments performed in this study, with assistance from RP, BR and RVHC. H-HH assisted with SARS-CoV-2 viral production. NP designed the mRNA vaccine. HM and PJCL produced the mRNA vaccine. GDV designed the *Ace2*™ allele and zygote targeting strategy. SN-H and MCCC conceptualized the work, designed all experiments and analyzed the data. SN-H, GDV and MCCC wrote the manuscript. All authors contributed to the article and approved the submitted version.

## Funding

This work was funded by Rockefeller University COVID-19 response grants to GDV and DM. SN-H is supported by a Bulgari Women & Science Fellowship. The funder was not involved in the study design, collection, analysis, interpretation of data, the writing of this article or the decision to submit it for publication. RVHC is supported by a long-term postdoctoral fellowship from Human Frontier Science Program (HFSP- LT000892/2020-L), MCCC is a Pew Latin American Fellow. DM is a Howard Hughes Investigator. GDV is a Burroughs-Wellcome Investigator in the Pathogenesis of Infectious Disease, a Pew-Stewart Scholar, and a MacArthur Fellow. CMR and H-HH were supported in part by the G. Harold and Leila Y. Mathers Charitable Foundation, the Bawd Foundation, Fast Grants, a part of Emergent Ventures at the Mercatus Center, George Mason University, and NIAID P01AI165075. NP was supported by the National Institute of Allergy and Infectious Diseases (R01AI146101 and R01AI153064).

## Acknowledgments

We thank the Rockefeller University staff for their invaluable support; T. Kurosaki (Osaka University) and T. Okada (RIKEN-Yokohama) for the S1pr2-CreERT2 strain.

## Conflict of interest

NP is named on a patent describing the use of nucleoside-modified mRNA in lipid nanoparticles as a vaccine platform. He has disclosed those interests fully to the University of Pennsylvania and has an approved plan in place for managing any potential conflicts arising from the licensing of that patent. PJCL is an employee of Acuitas Therapeutics, a company involved in the development of mRNA-LNP therapeutics. GDV is a scientific advisor for Vaccine Company, Inc.

The remaining authors declare that the research was conducted in the absence of any commercial or financial relationships that could be construed as a potential conflict of interest.

## Publisher’s note

All claims expressed in this article are solely those of the authors and do not necessarily represent those of their affiliated organizations, or those of the publisher, the editors and the reviewers. Any product that may be evaluated in this article, or claim that may be made by its manufacturer, is not guaranteed or endorsed by the publisher.
