# Supplementary material for: A minimally-edited mouse model for infection with multiple SARS-CoV-2 strains
Source: Front Immunol. 2022 Nov 14;13:1007080. doi: 10.3389/fimmu.2022.1007080 (PMC9703079; doi:10.3389/fimmu.2022.1007080)
Supplement: Supplementary file 1 [file DataSheet_1.pdf]

Supplementary Material

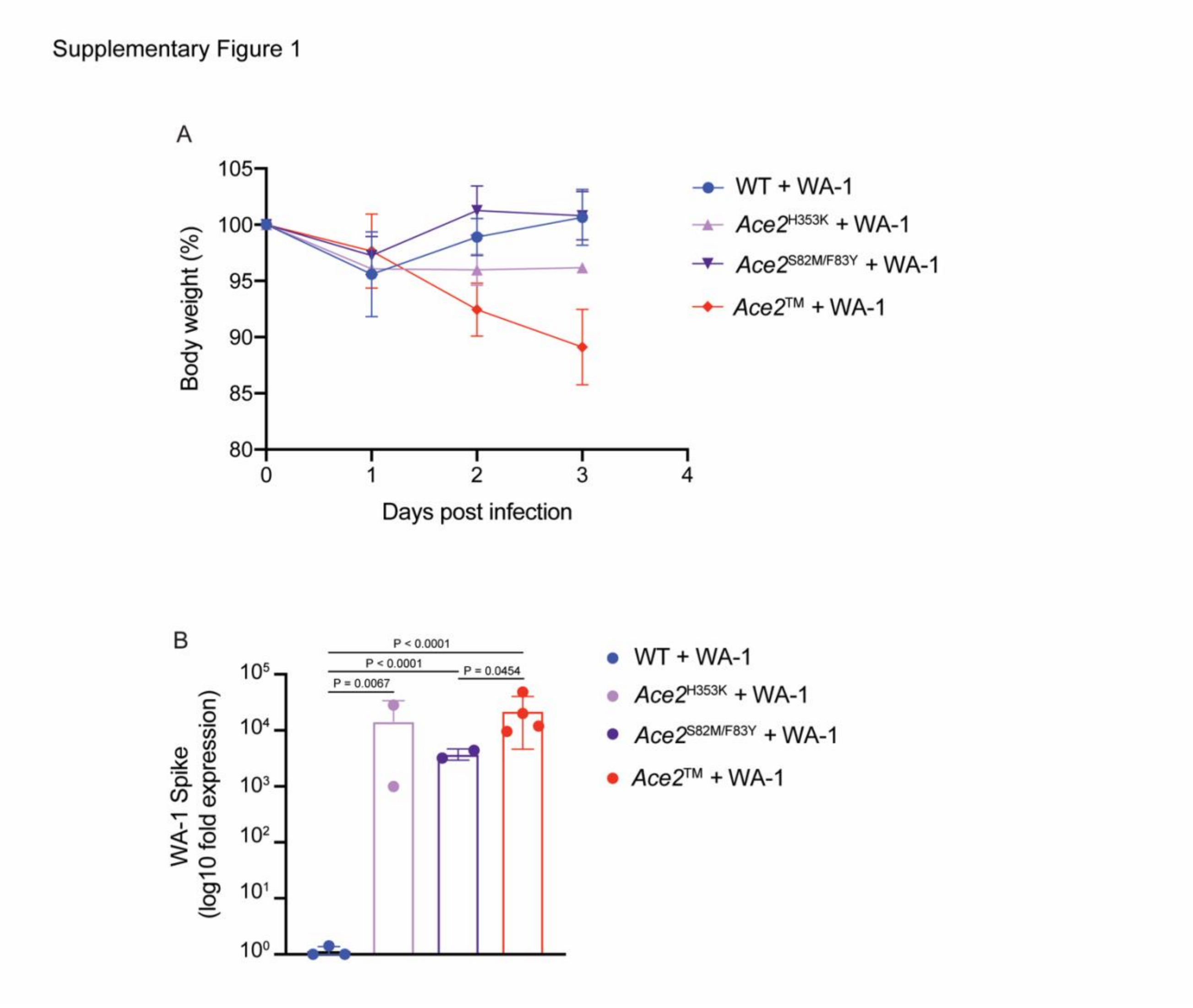

Supplementary Figure 1. Weight loss of different *Ace2*^{TM} mice infected with SARS-CoV-2 WA-1. (A) 8-14-week-old WT mice, *Ace2*^{H353K} mice, *Ace2*$^{S82M/F83Y}$ mice, and *Ace2*^{TM} mice were infected with WA-1 (2.34×10^5 PFU). Graph shows percent change in baseline body weight. **(B)** qRT-PCR of WA-1 *Spike* RNA loads in the lungs of mice at 3 dpi, shown as fold-change over RNA levels in WT infected mice. Total RNA input was normalized prior to qRT-PCR analysis.

Supplementary Figure 2

A Non-infected

	WT			
Area	1	2	3	4
Interstitial Congestion	0	0	0	0
Epithelial Damage	0	0	0	0
Inflammatory Infiltrate	0	1	0	0
Peribronchiolar Lymphocytic Inflammation	0	0	0	0
Haemorrhage	0	0	1	0
Interbronchiolar Macrophage	0	0	0	0
Total	0	1	1	0

B WA-1 infection

	WT								*Ace2* ™							
Area	1	2	3	4	5	6	7	8	1	2	3	4	5	6	7	8
Interstitial Congestion	1	1	1	1	1	1	1	1	1	1	1	2	1	1	2	1
Epithelial Damage	0	0	1	0	0	1	0	0	2	2	1	1	2	1	1	2
Inflammatory Infiltrate	1	1	1	1	2	1	2	1	1	2	1	1	2	1	2	2
Peribronchiolar Lymphocytic Inflammation	0	0	1	1	0	0	0	1	1	1	1	1	1	1	1	2
Haemorrhage	0	0	0	0	1	1	1	1	1	2	2	2	2	2	1	2
Interbronchiolar Macrophage	0	0	0	0	0	0	0	0	2	0	2	0	1	1	2	2
Total	2	2	4	3	4	4	4	4	8	8	8	7	9	7	9	11

C Omicron infection

	WT												*Ace2* ™											
Area	1	2	3	4	5	6	7	8	9	10	11	12	1	2	3	4	5	6	7	8	9	10	11	12
Interstitial Congestion	0	1	0	1	0	0	0	0	1	1	2	2	1	1	1	1	0	1	1	1	0	1	1	1
Epithelial Damage	0	0	0	0	0	0	0	0	0	0	0	0	0	0	0	1	1	1	1	1	1	1	0	0
Inflammatory Infiltrate	2	1	1	2	1	2	1	2	2	2	2	1	1	1	2	2	1	2	1	2	2	2	1	1
Peribronchiolar Lymphocytic Inflammation	1	1	1	1	1	1	1	1	1	1	2	2	1	1	1	1	1	1	1	1	1	1	1	1
Haemorrhage	1	1	1	1	1	2	1	2	2	2	2	2	1	1	2	2	1	1	1	1	2	2	2	2
Interbronchiolar Macrophage	0	0	0	0	0	0	0	0	0	0	0	0	0	0	1	0	0	1	0	1	0	1	1	0
Total	4	4	3	5	3	5	3	5	6	6	8	7	4	4	7	7	4	7	5	7	6	8	6	5

Supplementary Figure 2. Histopathology scoring sheet of lung specimens from WA-1 and Omicron infected *Ace2*™ and WT mice. Tissue pathology was assessed based on the presence of the indicated features in distinct areas from each lung specimen in **(A)** WT non-infected, **(B)** WA-1 infected WT and *Ace2*™ mice and **(C)** Omicron infected WT and *Ace2*™ mice.
